# Wetland Degradation and Ecological Restoration

**DOI:** 10.1155/2013/523632

**Published:** 2013-12-10

**Authors:** Junhong Bai, Baoshan Cui, Huicong Cao, Ainong Li, Baiyu Zhang

**Affiliations:** ^1^State Key Laboratory of Water Environment Simulation, School of Environment, Beijing Normal University, Beijing 100875, China; ^2^Northeast Institute of Geography and Agroecology, Chinese Academy of Sciences, Changchun 130102, China; ^3^Institute of Mountain Hazards and Environment, Chinese Academy of Sciences, Chengdu 610041, China; ^4^Faculty of Engineering and Applied Science, Memorial University of Newfoundland, St. John's, NL, Canada A1B 3X5

Wetlands are among the most important ecosystems on earth and functioned as the “kidneys” of the earth, which play an important role in maintaining ecological service functions. However, with the rapid growth in human populations, wetlands worldwide are suffering from serious degradation or loss as affected by wetland pollution, wetland reclamation, civilization and land use changes, and so forth. Wetland degradation has potential influences on human health, biodiversity, regional climate, and regional ecological security. Therefore, it is an urgent task to recover these degraded wetlands. In recent years, wetland protection, restoration, and its reasonable exploitation have been paid much more attention to by most governments and researchers. Moreover, wetland restoration has become the frontier fields of wetlands science, which has been listed as one of important themes in these recent international wetlands and ecological conferences. Understanding wetland degradation processes can contribute to better effective wetland restoration. Therefore, we organized this special issue on “wetland degradation and ecological restoration.” The objective of this special issue is to emphasize the effects of human activities on wetland ecosystems, the relationships between soil, water, and plant in wetlands, and wetland restoration issues and applications.

In total 25 submissions have been received from China and other countries until to the deadline date. However, only 11 papers were accepted to be published in this special issue on the basis of paper quality and reviewing process. The studied wetland types cover freshwater wetlands, coastal wetlands, lake wetlands, alpine wetlands and artificial wetlands. Although most contributors among these accepted manuscripts are from China except for 3 from Korea, France and Uganda, we still believe that this special issue can provide a platform to exhibit some related studies in such fields.

Among these accepted articles, Z. Luan et al. analyzed the response of *Carex lasiocarpa* in riparian wetlands to the environmental gradient of water depth by using the Gaussian model. Additionally, Luan et al. also showed readers the ecological impact of agricultural activity on marsh wetlands and assessed the functional efficacy. An ecological network for freshwater wetland conservation in the Central Yangtze Ecoregion was established and optimized based on large-scale gap analysis by X. W. Li et al. The polder effects of water table, residual available water capacity, and salt stress interdependence in coastal wetlands on sediment-to-soil conversion were investigated by R. Radimy et al. T. Seo et al. from Korea testified the functions of the littoral zone could be restored in an oligomesotrophic lake by installing an artificial vegetation island.

Q. Wang et al. reviewed the development of surface water quality models at three stages and gave some effective measures to standardize some surface water quality models. The eutrophication degree was also assessed in Baiyangdian Lake and the relationship between land use change and water quality was identified in Chaohu Lake by Wang et al. and Huang et al., respectively.

J. W. Kakuru et al. assessed the economic value of wetland resources and their contribution to food security in Uganda. A valuable way was established to overcome the gap that exists between the ability to construct computer simulation models to aid integrated land-use plan making and the demand for them by planning professionals by H. Yu et al. B. Kong and H. Yu developed an estimation model of freeze-thaw erosion and applied it to the Silingco Watershed Wetland of Northern Tibet.

Of course, the wetland degradation and ecological restoration not only contain the above published topics, but also include the following potential themes, for example, biogeochemical processes of carbon, nitrogen, and phosphorous in wetlands; wetland ecohydrological process and ecological water requirement; ecological risk assessment of wetland ecosystems; heavy metal pollution in wetland soils; wetland landscape changes and climate change and so forth. However, this special issue has no way to cover all these topics due to many objective causes. We really hope the future issues can improve them.

